# Inhibitory Effects of *Aucklandia lappa* Decne. Extract on Inflammatory and Oxidative Responses in LPS-Treated Macrophages

**DOI:** 10.3390/molecules25061336

**Published:** 2020-03-15

**Authors:** Jae Sung Lim, Sung Ho Lee, Sang Rok Lee, Hyung-Ju Lim, Yoon-Seok Roh, Eun Jeong Won, Namki Cho, Changju Chun, Young-Chang Cho

**Affiliations:** 1 Department of Biochemistry, Chonnam National University Medical School, Hwasun, Jeonnam-do 58128, Korea; dr.jslim7542@gmail.com; 2 Combinatorial Tumor Immunotherapy Medical Research Center, Chonnam National University Medical School, Hwasun, Jeonnam-do 58128, Korea; akira0128@naver.com; 3 Department of Molecular and Cellular Biology, Baylor College of Medicine, Houston, TX 77030, USA; puzim23@gmail.com; 4 ROK-Biotech, Jeollanamdo Biopharmaceutical Research Center, Hwasun, Jeollanam-do 58141, Korea; rok94@hanmail.net; 5 Department of Microbiology, Chonnam National University Medical School, Hwasun, Jeonnam-do 58128, Korea; 6 College of Pharmacy and Medical Research Center, Chungbuk National University, Cheongju 28160, Korea; ysroh@cbnu.ac.kr; 7 Department of Parasitology and Tropical Medicine, Chonnam National University Medical School, Hwasun, Jeonnam-do 58128, Korea; Parasite.woni@jnu.ac.kr; 8 College of Pharmacy, Chonnam National University, Gwangju 61186, Korea; cnamki@jnu.ac.kr

**Keywords:** *Aucklandia lappa* Decne. extract, lipopolysaccharide, nitric oxide, inducible nitric oxide, cyclooxygenase-2, nuclear factor-κB, mitogen-activated protein kinase, heme oxygenase-1, macrophage

## Abstract

*Aucklandia lappa* Decne., known as “Mok-hyang” in Korea, has been used for the alleviation of abdominal pain, vomiting, diarrhea, and stress gastric ulcers in traditional oriental medicine. We investigated the anti-inflammatory and antioxidative effects of the ethanol extract of *Aucklandia lappa* Decne. (ALDE) in lipopolysaccharide (LPS)-stimulated RAW 264.7 cells. ALDE significantly inhibited the LPS-induced nitric oxide (NO) production and reduced inducible nitric oxide synthase (iNOS) expression in RAW 264.7 cells. The production of other proinflammatory mediators, including COX-2, interleukin (IL)-6, IL-1β, and tumor necrosis factor (TNF)-α, was reduced by ALDE in LPS-stimulated RAW 264.7 cells. The mechanism underlying the anti-inflammatory effects of ALDE was elucidated to be the suppression of LPS-induced nuclear translocation of p65, followed by the degradation of IκB and the inhibition of the phosphorylation of mitogen-activated protein kinases (MAPK). In addition, ALDE showed enhanced radical scavenging activity. The antioxidant effect of ALDE was caused by the enhanced expression of heme oxygenase (HO-1) via stabilization of the expression of the nuclear transcription factor E2-related factor 2 (Nrf2) pathway. Collectively, these results indicated that ALDE not only exerts anti-inflammatory effects via the suppression of the NF-κB and MAPK pathways but also has an antioxidative effect through the activation of the Nrf2/HO-1 pathway.

## 1. Introduction

Inflammation is a central feature of various pathological conditions in the host defense against pathogens and in response to tissue injury. Macrophages are activated in response to various stimuli, such as LPS, and induce inflammation by producing inflammatory mediators, including nitric oxide (NO), prostaglandins (PGs), and proinflammatory cytokines, such as interleukin (IL)-1β, IL-6, and tumor necrosis factor (TNF)-α [1]. Although inflammation is important for the host defense against external stimuli, excess inflammation leads to severe immune disorders, such as septic shock, rheumatoid arthritis (RA), systemic lupus erythematosus (SLE), and inflammatory bowel disease (IBD) [2,3]. Thus, an agent that is able to alleviate the excessive inflammatory response may be a suitable candidate for the treatment of inflammatory disorders. Although a variety of anti-inflammatory drugs have been developed, including steroidal drugs and nonsteroidal anti-inflammatory drugs (NSAIDs), owing to the severe adverse effects of these drugs, natural products and their constituent compounds have been investigated for the development of new anti-inflammatory drugs.

Aucklandia lappa Decne., referred to as “Mok-hyang” in the 11th edition of the Korean Pharmacopoeia (KP11), is the root of Saussurea (Aucklandia) lappa Clarke (Chrysanthemum, Compositae). It contains approximately 1–2.5% of refined oils and has an abundance of sesquiterpenoid compounds (such as costunolide), which have many pharmacological effects, such as antibacterial [4] and anti-inflammatory [5] activity and an anti-inhibitory effect on vascular production [6]. Traditionally, “Mok-hyang” has been used for the treatment of vomiting, gastric pain, abdominal pain, anorexia, distension, and nausea [7]. Previously, it was reported that Aucklandia lappa Decne. has anti-ulcer [8], antiviral [9], and anticancer [10] effects. In addition, it has been reported that Aucklandia lappa Decne. extract (ALDE) inhibited inflammatory chemokine production in HaCaT cells [11] and exhibited anti-inflammatory effects in RAW 264.7 cells [12]. Thus, although the anti-inflammatory activity of ALDE has been reported, the mechanisms underlying these anti-inflammatory effects are not well elucidated. Herein, we investigated the anti-inflammatory and antioxidative effects of ALDE in LPS-stimulated macrophages and evaluated the associated molecular mechanism in vitro.

## 2. Materials and Methods

### 2.1. Extraction of ALDE

*Aucklandia lappa* Decne. was purchased from the Jeonnam Herb Medicine and Agriculture Cooperative (Hwasun, South Korea). Briefly, air-dried powdered (<0.2 mm) *Aucklandia lappa* Decne. (100 g) was extracted with 70% ethanol at approximately 70 °C for 9 h. The resultant ethanolic solution was filtered, evaporated, and freeze-dried to generate ALDE.

### 2.2. HPLC Chromatographic Analysis

Chromatographic analysis was performed on a reverse-phase Shimadzu HPLC system (Shimadzu Corp., Kyoto, Japan) with a Shimadzu LC-20AR solvent pump, coupled to a SPD-20A UV/VIS detector. Separation was performed on a Phenomenex C_18_ reverse-phase column (4.6 × 150 mm, 5 μm) using a gradient solvent system comprising acetonitrile (A) and water (B), with a composition by volume of 10% A at 0 min and 50% A at 40 min. The flow rate was 2 mL/min; the reaction was monitored spectrophotometrically at 254 nm. 

### 2.3. Cell Culture

RAW 264.7 cells (ATCC, Manassas, VA, USA), a mouse monocytic cell line, were maintained in Dulbecco’s modified Eagle’s medium supplemented with 10% fetal bovine serum (both from GE Healthcare Bio-Sciences, Pittsburgh, PA, USA), 50 U/mL penicillin, and 50 µg/mL streptomycin (Gibco; Thermo Fisher Scientific, Inc., Waltham, MA, USA) at 37 °C in humidified air containing 5% CO_2_.

### 2.4. Cell Viability Assay

RAW 264.7 cells (4 × 10^4^/well) were plated in 96-well plates. The cells were treated with various concentrations (1, 3, 5, 7, 9, 11, and 13 µg/mL) of ALDE for 24 h. Following treatment, cell viability was measured using an EZ-Cytox Cell Viability Assay kit (Daeil Lab Services Co., Ltd., Seoul, Korea). Briefly, the cells were incubated with the EZ-Cytox solution (containing a water-soluble tetrazolium salt) for 2 h at 37 °C. The absorbance of the supernatant at 450 nm was measured using a Synergy H1 Microplate Reader (BioTek Instruments, Inc., Winooski, VT, USA).

### 2.5. Measurement of NO Production

RAW 264.7 cells (4.0 × 10^4^ cells/well) were plated in 96-well plates. The cells were pretreated with various concentrations of ALDE (1, 2.5, 5, and 10 µg/mL) for 2 h and subsequently stimulated by LPS (0.5 µg/mL) for 24 h. The cell supernatants (100 µL) were transferred to new 96-well plates, and 100 µL Griess reagent (1% sulfanilamide, 0.1% N-1-naphthylethylenediamine dihydrochloride, and 2.5% phosphoric acid) was added. NaNO_2_ solutions (2.5, 5, 10, 25, 50, and 100 M) were used to generate a standard curve to calculate the concentration of NO in the supernatant. The absorbance at 540 nm was measured using a Synergy H1 Microplate reader (BioTek Instruments, Winooski, VT, USA).

### 2.6. RNA Preparation and cDNA Synthesis

RAW 264.7 cells (8.0 × 10^5^ cells/well) were seeded in 12-well plates. The cells were pretreated with various concentrations of ALDE (1, 2.5, 5, and 10 µg/mL) for 2 h and then stimulated with LPS (0.5 µg/mL) for 3 h. Total RNA was extracted using Accuzol (Bioneer Corporation, Daejeon, Korea) and synthesized into cDNA using a TOPscript cDNA synthesis kit in accordance with the manufacturer’s instructions.

### 2.7. Semiquantitative Reverse Transcription (RT)-PCR

The mixture for PCR was subjected to the following thermal profile: 17–25 cycles at 94 °C for 30 s, 60 °C for 30 s, and 72 °C for 30 s using a Bioer thermal cycler (Bioer Technology Co., Hangzhou, China). Following amplification, the PCR products (10 µL) were separated on a 1.5% (*w*/*v*) agarose gel and stained with ethidium bromide. The following primers were used: Mouse iNOS (sense, 5′-GCA TGGAACAGTATAAGGCAAACA-3′; antisense, 5′-GTTTCTGGTCGATGTCATGAGCAA-3′), COX-2 (sense, 5′-GCATGGAACAGTATAAGGCAAACA-3′; antisense, 5′-GTTTCTGGT CGATGTCATGAGCAA-3′), TNF-α (sense, 5′-GTGCCAGCCGATGGGTTGTACC-3′; antisense, 5-′AGGCCCACAGTCCAGGTCACTG-3′), IL-6 (sense, 5′-TCTTGGGACTGATG CTGGTGAC-3′; antisense, 5′-CATAACGCACTAGGTTTGCCGA-3′), IL-1β (sense, 5′-AGC TGTGGCAGCTACCTGTG-3′; antisense, 5′-GCTCTGCTTGTGAGGTGCTG-3′), and GAPDH (sense, 5′-GTCTTCACCACCATGGAGAAGG-3′; antisense, 5′-CCTGCTTCACCA CCTTCTTGCC-3′).

### 2.8. Western Blotting

The whole-cell lysate was prepared by incubating the cells in a RIPA buffer (50 mM Tris-HCl pH 8.0, 150 mM NaCl, 0.1% SDS, 0.5% deoxycholate, 1% NP-40, and 1 mM EDTA) with protease inhibitors (XXX) for 30 min at 4 °C followed by centrifugation (13,200 rpm for 15 min). The supernatant was denatured in 5× SDS sample buffer (200 mM Tris-HCl pH 6.8, 40% glycerol, 8% SDS, 200 mM dithiothreitol, and 0.08% bromophenol blue) at 95 °C for 5 min, separated by SDS-PAGE, and then transferred to nitrocellulose membranes. To block nonspecific binding, we incubated the membranes in 5% nonfat dry milk in Tris-buffered saline and Tween-20 (25 mM Tris-HCl pH 8.0, 125 mM NaCl, and 0.5% Tween-20) for 1 h at RT. The membranes were incubated with primary antibodies at 4 °C overnight and then incubated with horseradish peroxidase-conjugated (HRP)-conjugated secondary antibodies for 1 h at RT. Pierce ECL Western blotting substrate for enhanced chemiluminescence (Thermo Fisher Scientific, Inc.) was used to detect the HRP-conjugated secondary antibodies. Protein expression was analyzed and quantified using LabWorks software version 4.6 (UVP, LLC; Analytik Jena AG, Upland, CA, USA). 

### 2.9. ELISA

RAW 264.7 cells (4.0 × 10^4^ cells/well) were plated in 96-well plates. The cells were pretreated with various concentrations of ALDE (1, 2.5, 5, and 10 µg/mL) for 2 h and then stimulated with LPS (0.5 µg/mL) for 24 h. The expression of the indicated cytokines in the cell supernatant was measured using an ELISA kit in accordance with the manufacturer’s instructions. Briefly, the culture plates were incubated overnight with a coating solution at 4 °C, washed three times with 1× PBS/0.05% Tween-20 (PBST), and then incubated with 1× assay diluent (from the ELISA kit) for 1 h at RT. The supernatants and standard solutions were incubated for 2 h at RT and then washed three times. Next, the plate was incubated with Ab Detection solution (also from the ELISA kit) for 1 h at RT and then washed three times. Subsequently, the plate was incubated with a horseradish peroxidase-streptavidin solution for 30 min at RT and then washed five times. Finally, the plate was incubated with a solution of 3,3′,5,5′-tetramethylbenzidine for 10 min in the dark; then, 1 N H_3_PO_4_ was added to stop the reaction. The absorbance at 450 nm was measured spectrophotometrically using a Synergy H1 Microplate reader.

### 2.10. Subcellular Fractionation

Subcellular fractionation was performed as described previously [13]. Briefly, the cells were washed twice with ice-cold PBS and lysed with 200 μL of cytoplasmic lysis buffer (10 mM HEPES, 60 mM KCl, 1 mM EDTA, 1 mM DTT, and 1 mM PMSF) on ice for 15 min; subsequently, 10 μL of 0.075% (*v*/*v*) IGEPAL CA-630 (Sigma-Aldrich, St. Louis, MO, USA) was added. After brief centrifugation (10 s), the supernatants were collected for the cytoplasmic fraction. Next, the pellet was resuspended in 25 μL of a nuclear extraction buffer (20 mM Tris Cl, 420 mM NaCl, 1.5 mM MgCl_2_, 0.2 mM EDTA, 1 mM PMSF, and 25% (*v*/*v*) glycerol) on ice for 30 min and vortex mixed every 10 min. After centrifugation for 30 min at 4 °C, the supernatant was collected to obtain the nuclear fraction. Western blotting was performed using anti-α-tubulin (cytoplasm) and anti-lamin B1 (nucleus) antibodies to confirm the cytoplasmic and nuclear extracts, respectively.

### 2.11. DPPH Free Radical Scavenge Activity

ALDE ethanolic solution was mixed with the same volume of 0.4 mM DPPH ethanolic solution. The mixture was allowed to react at RT in the dark for 10 min. The absorbance at 517 nm was measured using a Synergy H1 Microplate reader. The free radical scavenging activity was calculated as a percentage using the following equation [14].

DPPH free radical scavenging activity (%) = [1 − (A_sample_/A_blank_)] × 100.

### 2.12. Statistical Analysis

The data are presented as the mean ± standard error of the mean. Multiple experimental groups were compared by one-way analysis of variance followed by Dunnett’s post-hoc test calculated using GraphPad Prism (version 3.0; GraphPad Software, Inc., La Jolla, CA, USA); *p*-values < 0.05 were considered statistically significant.

## 3. Results

### 3.1. HPLC and Costunolide-Related Results

**Figure 1:**
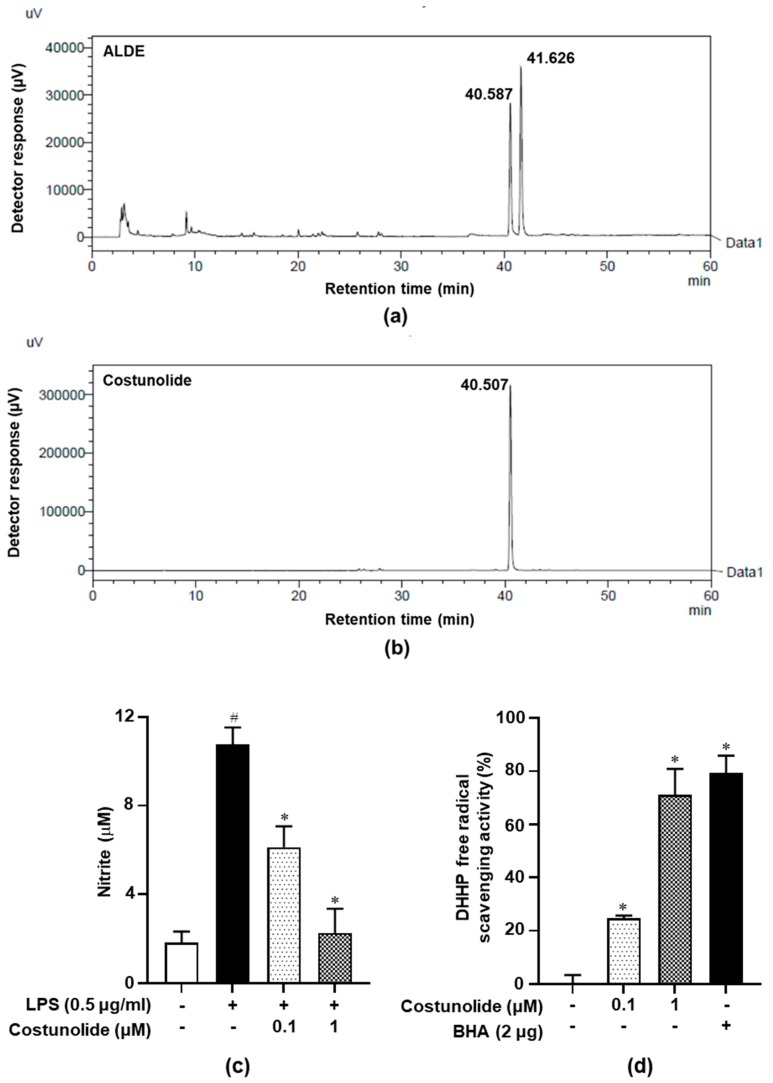
HPLC analysis of the ethanol extract of *Aucklandia lappa* Decne. (ALDE) and costunolide. The phytochemical characteristics of (**a**) ALDE and its major component, (**b**) costunolide, were analyzed using HPLC. (**c**,**d**) NO inhibitory effect of costunolide. Data represent the mean ± SEM of three independent experiments. ^#^ *p* < 0.05 vs. LPS-untreated control group; ∗ *p* < 0.05 vs. LPS-treated group. (**d**) DPPH radical scavenging activity of costunolide. Data represent the mean ± SEM of three independent experiments. ∗ *p* < 0.05 vs. untreated group. ALDE: Ethanol extract of *Aucklandia lappa* Decne.; LPS: Lipopolysaccharide; NO: Nitric oxide.

Before the investigation of the effects of ALDE on inflammation and oxidative stress, an evaluation of the major components that exhibit anti-inflammatory and antioxidative effects was required. Costunolide, a component of ALDE, has been known to inhibit the production of inflammatory mediators and enhance HO-1 expression [15]. HPLC analysis was performed to show that ALDE and costunolide exhibited the same retention time. As shown in Figure 1a,b, HPLC analysis of costunolide showed a single peak at 40.507 min. One of the major peaks of the ALDE HPLC data (40.587 and 41.626 min) has the same retention time as costunolide. As previously reported, costunolide inhibited LPS-induced NO production in RAW 264.7 cells and exhibited significant radical scavenging activity compared with BHA, a positive control (Figure 1c,d). These results indicated that costunolide was a major component of ALDE and led us to study the anti-inflammatory effects and underlying regulatory mechanism of action of ALDE in murine macrophages. 

### 3.2. ALDE Suppressed the Release of NO in LPS-Stimulated RAW 264.7 Cells

**Figure 2:**
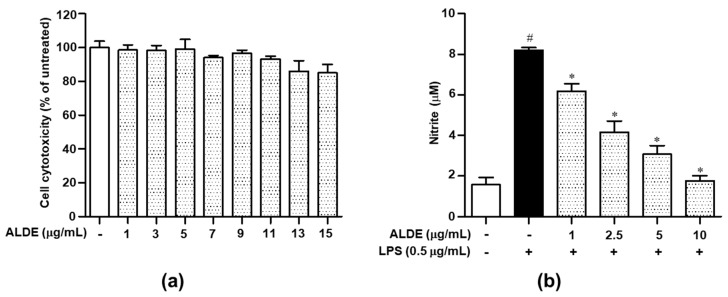
Effects of ALDE on cell viability and NO production in RAW 264.7 cells. (**a**) RAW 264.7 cells were treated with various concentrations of ALDE for 24 h. Subsequently, cell viability was measured using the EZ-Cytox reagent and compared with that in the untreated group. (**b**) RAW 264.7 cells were treated with LPS (0.5 μg/mL) in the presence of ALDE (1, 2.5, 5, and 10 μg/mL) for 24 h. Subsequently, NO production in the culture supernatant was measured using a Griess assay. NO secretion was calculated using a standard curve of concentrations of nitrite standard solution. The data presented are the mean ± SEM of three independent experiments. Differences between groups were analyzed using the Mann–Whitney *U* test. ^#^ *p* < 0.05 vs. LPS-untreated control groups; ^∗^ *p* < 0.05 vs. LPS-treated groups. ALDE: Ethanol extract of *Aucklandia lappa* Decne.; LPS: Lipopolysaccharide; NO: Nitric oxide.

To investigate the effect of ALDE on the viability of RAW 264.7 cells, we treated the cells with the indicated concentrations of ALDE for 24 h and then quantified the metabolic conversion of a tetrazolium salt to a formazan dye to determine the percentage of viable cells. ALDE exerted no significant cytotoxicity in RAW 264.7 cells at concentrations below 13 μg/mL (Figure 2a). Therefore, subsequent experiments were performed at ALDE concentrations of 1, 2.5, 5, and 10 μg/mL, which were known to not exert cytotoxic effects. To investigate the anti-inflammatory effects of ALDE, we examined the effect of ALDE on the production of NO, a well-known proinflammatory mediator, in LPS-stimulated RAW 264.7 cells. The nitrite level in the culture medium of the RAW 264.7 cells was significantly increased upon LPS treatment. However, in the cells pretreated with ALDE, a considerable dose-dependent suppression of LPS-induced NO production was observed (Figure 2b). These results suggested that ALDE markedly reduced the NO production in LPS-stimulated RAW 264.7 cells.

### 3.3. ALDE Inhibited the Expression of Proinflammatory Enzymes, iNOS and COX-2, in LPS-Stimulated RAW 264.7 Cells 

**Figure 3:**
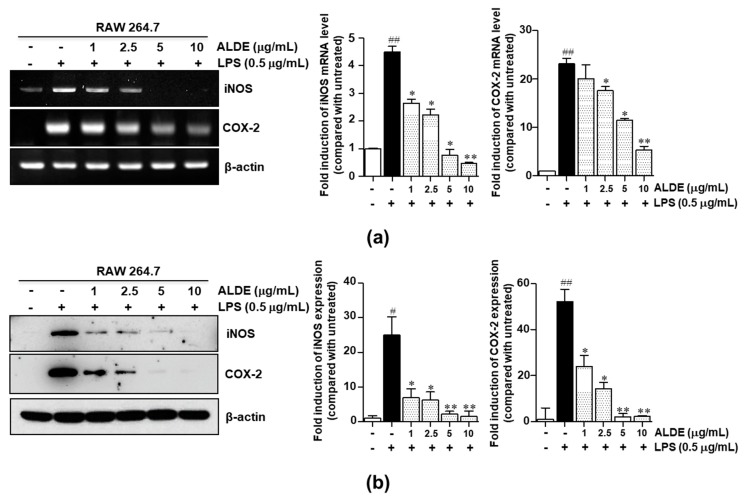
Effects of ALDE on the expression of iNOS and COX-2. RAW 264.7 cells were treated simultaneously with LPS and ALDE (1, 2.5, 5, and 10 μg/mL). (**a**) Following stimulation for 6 h, total RNA was extracted and reverse transcribed to cDNA. mRNA expression of *iNOS* and *COX-2* was analyzed by RT-PCR. (**b**) After stimulation for 24 h, the total protein was extracted. The protein expression of iNOS and COX-2 was detected by Western blotting. The protein β-actin was used as a loading control for both RT-PCR and Western blotting. The relative density of the mRNA or protein expression was normalized to that of β-actin and is presented in quantitative graphs. The data presented are the mean ± SEM of three independent experiments. Differences between groups were analyzed using the Mann–Whitney *U* test. ^#^ *p* < 0.05, ^##^ *p* < 0.01 vs. LPS-untreated control groups; ∗ *p* < 0.05, ∗∗ *p* < 0.01 vs. LPS-treated groups. COX-2: Cyclooxygenase-2; iNOS: Inducible nitric oxide synthase.

The expression of proinflammatory enzymes, including COX-2 and iNOS, plays an important role in the immune response from activated macrophages through the production of NO and PGE2, respectively [16,17]. We investigated the effect of ALDE on the expression of iNOS and COX-2 in LPS-stimulated RAW 264.7 cells. As shown in Figure 3, the expression of iNOS and COX-2 was increased markedly in response to LPS treatment. When RAW 264.7 cells were treated with various concentrations of ALDE, the LPS-induced expression of iNOS and COX-2 was significantly decreased in a dose-dependent manner (Figure 3a,b). These results indicated that ALDE inhibited the production of proinflammatory mediators through the inhibition of the expression of their responsible enzymes, iNOS and COX-2.

### 3.4. ALDE Inhibited the Production of Proinflammatory Cytokines in LPS-Stimulated Macrophages 

**Figure 4:**
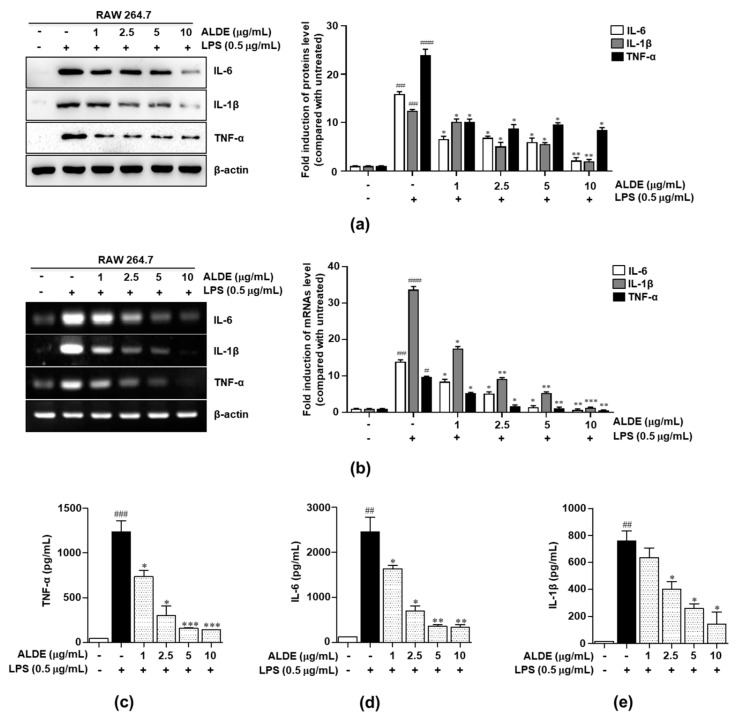
Inhibitory effects of ALDE on the production of proinflammatory cytokines. RAW 264.7 cells were treated with LPS in the presence of ALDE (1, 2.5, 5, and 10 μg/mL). (**a**) After stimulation for 24 h, the total cellular proteins were extracted. The expression of interleukin (IL)-6, IL-1β, and tumor necrosis factor (TNF)-α was detected by Western blotting. (**b**) After simulation for 6 h, total RNA was extracted. The mRNA expression of IL-6, IL-1β, and TNF-α was analyzed by RT-PCR. β-Actin was used as a loading control for both RT-PCR and Western blotting. The relative density of the mRNA or protein expression was normalized to that of β-actin and is presented in quantitative graphs. (**c**–**e**) After stimulation for 24 h, the culture supernatants were collected and analyzed for IL-6, IL-1β, and TNF-α production by ELISA. The data presented are the mean ± SEM of three independent experiments. Differences between groups were analyzed using the Mann–Whitney *U* test. ^#^ *p* < 0.05, ^##^ *p* < 0.01, ^###^ *p* < 0.001 vs. LPS-untreated control groups; ∗ *p* < 0.05, ∗∗ *p* < 0.01, ∗∗∗ *p* < 0.001 vs. LPS-treated groups. IL: Interleukin; TNF: Tumor necrosis factor.

To investigate whether ALDE affected the expression of proinflammatory cytokines, we performed Western blotting analysis and RT-PCR. The expression of IL-6, IL-1β, and TNF-α was significantly increased after treatment with LPS but markedly decreased in a dose-dependent manner after pretreatment with ALDE (Figure 4a,b). To confirm the inhibitory effect of ALDE on the cytokine production induced by LPS stimulation, we used ELISA. As shown in Figure 4c–e, LPS-stimulated RAW 264.7 cells treated with ALDE exhibited concentration-dependent inhibition of the proinflammatory cytokines, such as TNF-α, IL-6, and IL-1β. These results suggested that ALDE exerted anti-inflammatory effects through the inhibition of proinflammatory cytokines.

### 3.5. ALDE Suppressed Both NF-κB Activation and MAPK Phosphorylation in LPS-Stimulated Macrophages 

**Figure 5:**
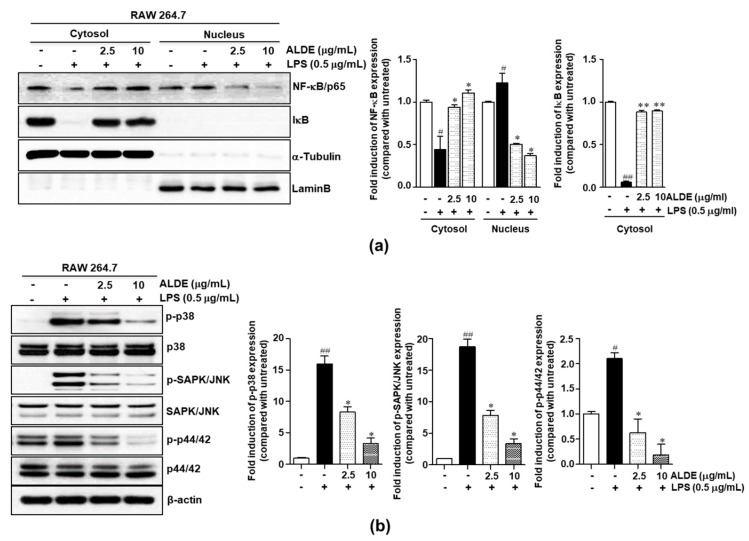
Inhibitory effects of ALDE on the nuclear translocation of NF-κB and the MAPK signaling pathway. RAW 264.7 cells were pretreated with ALDE (0, 2.5, and 10 μg/mL) for 2 h and then stimulated with LPS (0.5 μg/mL) for 15 min. Cytosolic extracts and nuclear extracts were prepared. (**a**) The expression of NF-κB/p65 and IκB was detected by Western blotting; α-tubulin was used as a cytosolic loading control and Lamin B was used as a nuclear loading control. (**b**) The expression of the proteins associated with the MAPK signaling pathway (p38, p44/42 ERK, and JNK) was detected by Western blotting, with β-actin used as a loading control. The relative density of the protein expression was normalized to each loading control and is presented in quantitative graphs. The data presented are the mean ± SEM of three independent experiments. Differences between groups were analyzed using the Mann–Whitney *U* test. ^#^ *p* < 0.05, ^##^ *p* < 0.01 vs. LPS-untreated control groups; ∗ *p* < 0.05, ∗∗ *p* < 0.01 vs. LPS-treated groups. NF-κB: Nuclear-κB; IκB: Inhibitor of κB; MAPK: Mitogen-activated protein kinase; p-: Phosphorylated; SAPK/JNK: Stress-associated protein kinase/c-Jun N-terminal kinase; ERK: Extracellular signal-regulated kinase.

The NF-κB and MAPK signaling pathways are the major regulators of the expression of inflammatory mediators [18,19]. To elucidate the mechanisms underlying the anti-inflammatory effects of ALDE, we examined the changes in NF-κB translocation into nucleus after treatment with ALDE. As shown in Figure 5a, the LPS-induced degradation of IκB was significantly suppressed by ALDE treatment in the cytosolic fraction. In contrast, the level of LPS-induced nuclear NF-κB/p65 protein, which is translocated into nucleus after IκB degradation, was decreased by ALDE treatment in RAW 264.7 cells (Figure 5a). Next, we investigated whether ALDE regulated the LPS-induced phosphorylation of MAPKs. As shown in Figure 5b, LPS treatment significantly induced the phosphorylation of p38, JNK, and ERK, although ALDE significantly suppressed the phosphorylation of these proteins in a dose-dependent manner. These results suggested that the anti-inflammatory effects of ALDE were mediated by the inhibition of the activation of both NF-κB and MAPK signaling.

### 3.6. ALDE Increased the Expression of HO-1 and the Nuclear Translocation of Nrf2 in LPS-Stimulated Macrophages 

**Figure 6:**
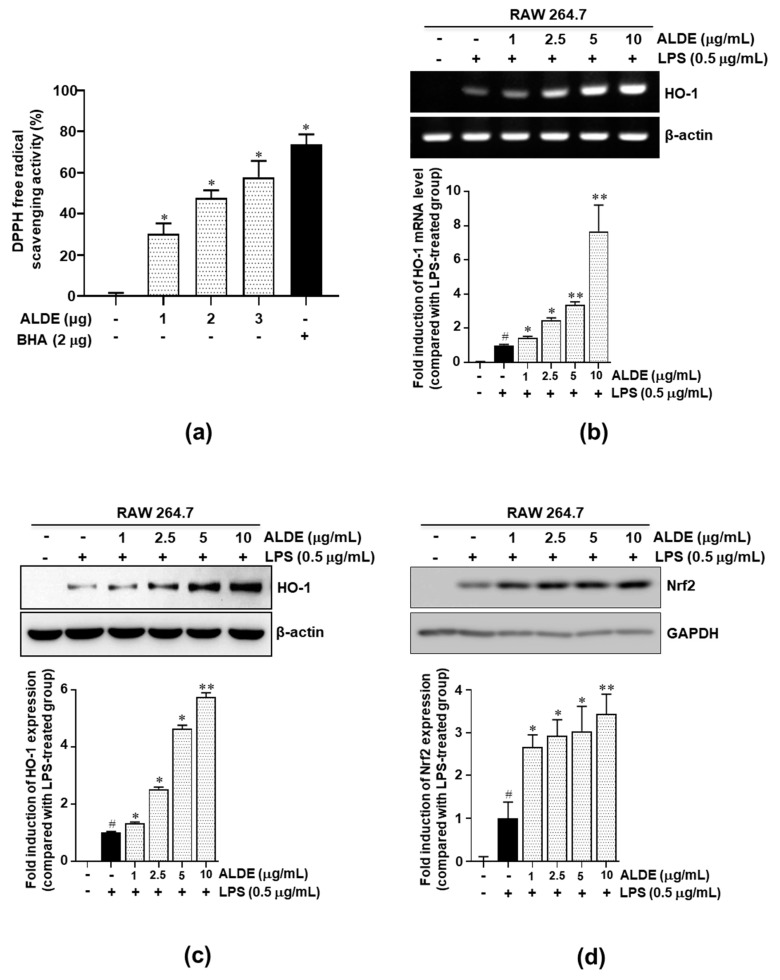
Antioxidative effects of ALDE. (**a**) DPPH free radical scavenging activity is represented as the mean ± SEM. ∗ *p* < 0.01 relative to the MeOH-reacted group. BHA was used as a positive control. (**b**–**d**) RAW 264.7 cells were treated with LPS in the presence of ALDE (1, 2.5, 5, and 10 μg/mL). (**b**) After stimulation for 6 h, total RNA was extracted. HO-1 mRNA expression was analyzed by RT-PCR. (**c**,**d**) After stimulation for 24 h, total protein was extracted. The protein expression of HO-1 (**c**) and Nrf2 (**d**) was detected by Western blotting. The relative density of the mRNA and protein expression was normalized to that of β-actin and is presented by quantitative graphs. The data presented are the mean ± SEM of three independent experiments. Differences between groups were analyzed using the Mann–Whitney *U* test. ^#^ *p* < 0.05 vs. LPS-untreated control groups; ^∗^ *p* < 0.05, ^∗∗^ *p* < 0.01 vs. LPS-treated groups. Nrf2: Nuclear factor erythroid 2-related factor; HO-1: Heme oxygenase-1.

To investigate whether ALDE exhibited antioxidative effects, we assayed the radical scavenging activity. As shown in Figure 6a, ALDE showed significant radical scavenging activity compared with BHA, a positive control. As the antioxidative effects were mediated by antioxidative regulators, such as HO-1, the profile of ALDE-mediated HO-1 expression was investigated in LPS-stimulated RAW 264.7 macrophages. Both mRNA expression and protein expression of HO-1 in LPS-stimulated RAW 264.7 cells were significantly increased by ALDE treatment in a dose-dependent manner (Figure 6b,c). As Nrf2 is a major regulator of the expression of HO-1 [20], we investigated whether ALDE enhanced the stability and, subsequently, the expression of Nrf2. We found that the expression of Nrf2 was increased by ALDE treatment (Figure 6d). These data suggested that ALDE exerts antioxidative effects through the activation of the Nrf2/HO-1 pathway.

## 4. Discussion

Recently, natural products have been considered important sources of drugs targeting a variety of diseases, such as cancer and inflammatory disorders [21,22,23,24]. In particular, many researchers have reported that the extracts of natural products, such as fruits, vegetables, plants, and their formulations have significant anti-inflammatory effects [25,26,27,28,29,30]. Similarly, as costunolide is a naturally occurring sesquiterpene lactone that has been extensively studied for its anti-inflammatory activity and is one of major components of ALDE, we investigated the anti-inflammatory effects of ALDE and the underlying mechanism of action [15,31,32]. 

The expression of iNOS is stimulated by not only proinflammatory cytokines, such as TNF-α, IL-1β, and IL-6 [33], but also bacterial products such as LPS [34]. Therefore, the inhibitory effects of natural products on NO production that occurs through the inhibition of iNOS expression, suggest that they may be potent drug candidates for the treatment of inflammatory diseases. TNF-α antagonists, including anti-TNF receptor antibodies and anti-IL-6 receptor antibodies, are currently used to inhibit the action of each proinflammatory cytokine for the treatment of RA and Crohn’s disease [35,36]. Thus, agents that inhibit these proinflammatory cytokines have been suggested as therapeutic candidates for the treatment of immune diseases. In this study, we examined the inhibitory effects of ALDE on the production of various LPS-stimulated proinflammatory mediators in RAW 264.7 cells and found that ALDE significantly inhibited the production of these mediators (Figure 4). These results suggested that ALDE is a potent anti-inflammatory agent and exerts this action through the inhibition of proinflammatory responses.

HO-1 expression is enhanced by various proinflammatory stimulators, such as NO, LPS, cytokines, and other oxidants [17,20,37]. Previous studies have shown that the induction of HO-1 can represent an efficient antioxidant system and a potential pharmacological target in a variety of oxidant- and inflammatory-mediated diseases [38,39,40] and that this was involved in the inhibitory effects on LPS-induced NO production [41]. In this study, we observed that LPS itself caused a slight increase in HO-1 expression and that ALDE further enhanced HO-1 expression in LPS-stimulated RAW 264.7 macrophages. These results suggested that the increase in HO-1 expression induced by ALDE could inhibit NO production in LPS-stimulated RAW 264.7 cells.

The multifunctional regulator nuclear factor erythroid 2-related factor (Nrf2) is considered a cytoprotective factor that regulates the expression of genes coding for antioxidant, anti-inflammatory, and detoxifying proteins [20]. The major roles of Nrf2 are mediated by Nrf2-dependent genes and their encoded proteins, including HO-1, which have important roles in the removal of toxic heme, producing biliverdin, iron ions, and carbon monoxide. HO-1 and its products exert beneficial effects by protecting cells from oxidative injury, apoptosis, and inflammation [39]. Thus, the activation of the Nrf2 pathway is a possible explanation for the increase in HO-1 expression. As the nuclear translocation of Nrf2 allows the activation of the transcription of the HO-1 gene [20], we examined whether ALDE induced the nuclear translocation of Nrf2 in LPS-stimulated RAW 264.7 cells. We found that ALDE stabilized the Nrf2 protein expression (Figure 6d). These results suggested that the increase in HO-1 expression induced by ALDE may be mediated via the Nrf2 pathway. 

In conclusion, we showed that ALDE significantly suppressed the production of NO and inhibited the expression of iNOS, COX-2, and proinflammatory cytokines in LPS-stimulated murine macrophages. The inhibitory effect was mediated by the inhibition of NF-κB translocation and MAPK phosphorylation. Moreover, we found that ALDE induced the expression of HO-1 and increased the nuclear translocation of Nrf2 in LPS-stimulated macrophages. Collectively, our results suggested that ALDE may exert potent therapeutic effects in various inflammatory diseases.
